# Thermomechanical load in a nonlocal rotating magneto-thermoelastic orthotropic material with Green Naghdi-III model

**DOI:** 10.1038/s41598-026-40500-y

**Published:** 2026-04-09

**Authors:** Doaa. M. Salah, A. M. Abd-Alla, S. M. M. El-Kabeir, F. S. Bayones

**Affiliations:** 1 Department of Mathematics, Faculty of Science, Aswan University, Aswan, Egypt; 2 Department of Mathematics, Faculty of Science, Sohag University, Sohag, Egypt; 3 Department of Mathematics and Statistics, College of Science, Taif University, P.O.Box 11099, Taif, 21944 Saudi Arabia

**Keywords:** Nonlocal thermoelastic, Orthotropic material, Thermomechanical, Magnetic field, Rotation, Normal mode analysis, Engineering, Materials science, Mathematics and computing, Physics

## Abstract

The objective of this study is to examine the influence of nonlocal thermoelasticity parameters on an orthotropic medium subjected to magnetic fields and rotational effects, within the framework of Green-Naghdi thermoelasticity theory (Type III). A time-dependent thermal load is applied to the free surface of the medium, and analytical solutions for the resulting thermal stresses, displacement, and temperature fields are derived using the normal mode analysis and eigenvalue approach techniques. Numerical simulations, implemented through MATHEMATICA programming, are conducted for a representative material to validate the theoretical model. The results are presented graphically to highlight the effects of various parameters, including time, non-locality, magnetic intensity, and rotational speed, on the thermoelastic response. These findings offer significant insights for advanced engineering and scientific applications, especially in geophysics, aerospace, and biomedical engineering, where complex multiphysical interactions and nonlocal effects play a critical role. The study also contributes to the ongoing development of generalized thermoelastic models capable of accurately capturing wave propagation and heat conduction behaviours in anisotropic and heterogeneous materials.

## Introduction

In recent years, the study of thermomechanical interactions in advanced materials has gained significant traction across various engineering and scientific disciplines. This interest is largely driven by the increasing demand for high-performance materials that can operate reliably under extreme conditions, such as those encountered in aerospace structures, nuclear reactors, microelectromechanical systems (MEMS), and other high-temperature or high-speed environments. Among such materials, orthotropic media characterized by direction-dependent mechanical and thermal properties have emerged as critical components in modern engineering applications due to their anisotropic behavior, which offers enhanced performance when appropriately exploited. The study of thermomechanical behavior in advanced materials has become increasingly vital due to the growing demand for precision in high-performance engineering applications. Among such materials, orthotropic media characterized by directional dependence of mechanical properties are frequently encountered in aerospace, civil, and mechanical engineering. When these materials operate under the influence of complex physical fields such as magnetic and rotational effects, their response to thermal and mechanical loads becomes considerably intricate. In recent years, the incorporation of nonlocal elasticity theories has garnered significant attention for capturing size-dependent behavior in microscale and nanoscale structures, which classical continuum theories fail to address. Additionally, the Green–Naghdi theory of thermoelasticity, particularly the Type III model, offers a more accurate depiction of heat conduction without energy dissipation, aligning well with the principles of modern thermomechanical modeling.

Simultaneously, the influence of nonlocal elasticity theory has become increasingly relevant in modeling materials at micro- and nano-scales, where classical continuum mechanics fails to accurately predict the response due to size-dependent effects. By incorporating long-range interatomic interactions, nonlocal theories provide a more realistic representation of material behavior at small scales. This is particularly important when considering high-frequency dynamic loading or when the characteristic length scale of the deformation approaches the material’s microstructure. Adding another layer of complexity, the presence of magnetic fields can drastically alter the mechanical and thermal response of conductive materials. Magneto-thermoelastic interactions introduce Lorentz forces and electromagnetic coupling effects, which significantly affect wave propagation, stress distribution, and thermal gradients within the medium. When such a system is subjected to rotational motion, gyroscopic forces and Coriolis effects further influence the dynamics, introducing additional challenges in both analytical and numerical modeling. To capture the full spectrum of thermomechanical behavior under these multifaceted influences, this study employs the Green–Naghdi Type III (GN-III) model a thermodynamic framework that accommodates finite speed heat conduction without energy dissipation. Unlike the classical Fourier model or earlier Green–Naghdi formulations, the GN-III model presents a more physically realistic approach by allowing for the propagation of thermal waves with finite speed, a phenomenon often observed in low-temperature environments or high-rate thermal loading scenarios. In this context, the present work aims to explore the intricate behavior of a nonlocal orthotropic medium under the combined effects of thermal, magnetic, and rotational fields using the GN-III model. The analysis focuses on the propagation of thermomechanical disturbances, evaluating how nonlocality, anisotropy, magnetic field strength, and angular velocity influence the stress, displacement, and temperature distributions over time. Through this comprehensive study, we seek to provide deeper insights into the coupled magneto-thermoelastic behavior of advanced materials, offering theoretical foundations that may guide the design and optimization of next-generation devices and systems. Biot^1^ laid the foundation for coupled thermoelasticity by introducing a revolutionary theory that integrated thermal and elastic effects. He derived the heat conduction equation and proposed the classical dynamic (CD) theory, grounded in Fourier’s heat law, to describe the swift propagation of thermal waves. To tackle the limitations of traditional models, Biot^1^ proposed the theory of coupled thermoelasticity, which incorporates both thermal and mechanical interactions. This theory led to the development of the CD model, based on Fourier’s law, to study rapid thermal wave propagation. Building on Biot’s framework, Lord and Shulman (LS)^2^ introduced a notable enhancement by incorporating a single relaxation time into the heat conduction equation, allowing for finite speed of thermal wave propagation. In parallel, Green and Lindsay (GL)^3^ expanded the theory further by including two relaxation times, giving rise to a more comprehensive model of generalized thermoelasticity. Green and Naghdi^4^ proposed three different types of generalized thermoelastic theories (Type I, II, and III) in the 1990s, aiming to extend classical thermoelasticity beyond Fourier’s law, which implies infinite speed of heat propagation. The Type III model is the most general and physically rich among them. The advancements introduced by LS, GL and GN (I, II, III) and others marked a significant shift in thermoelastic theory, leading to generalized models that account for thermal wave delays and have been extensively investigated in subsequent research^5–12^. Thermal shock refers to the sudden application of a thermal load typically a rapid increase or decrease in temperature that can cause severe mechanical stress in a solid material. In thermoelasticity, thermal shock is analysed to understand how materials respond to such abrupt temperature changes, especially in terms of stress distribution, deformation, and potential failure. This has received wide attention from researchers who have studied its behavior on the generalized thermodynamic theory of various elastic materials, taking into account some other influences within the framework of different models^13,14^. In classical thermoelasticity, the response at a given point in a material depends only on the local state variables such as temperature, displacement, and stress at that point. However, in nonlocal thermoelasticity, the material response at a point is influenced by the behavior of surrounding points within a certain neighbourhood. This is especially important for micro- and nanoscale materials, where size effects become significant. It defines how much the stress or strain at a point depends on the fields in the surrounding region. The foundational framework for nonlocal thermoelasticity was established by Eringen^15^, who introduced a theory accounting for long-range interatomic forces in continuous media. Building on Eringen’s nonlocal theory, recent studies have explored more complex material behaviors. For instance, Othman et al.^16–18^ investigated the impact of nonlocal effects on a poro-thermoelastic solid with temperature-dependent properties using the three-phase-lag model. Salah et al.^19^ investigated the effects of magnetic fields and initial stress on wave propagation in a rotating photothermal semiconductor medium subjected to ramp-type heating and internal heat generation, providing insights into thermoelastic behavior under complex physical influences. The influence of magnetic fields, initial stress, and internal heat sources on photothermal semiconductor media has been rigorously examined by Salah et al.^19^, emphasizing the role of rotational effects under ramp-type thermal loading. In a related study, Abo-Dahab et al.^20^ analyzed wave propagation phenomena induced by electromagnetic and pulse laser heating during electron and hole excitation in a rotating semiconductor, highlighting the intricate interplay between thermal, electromagnetic, and mechanical fields. Expanding on this, Abo-Dahab et al.^20^ explored the excitation dynamics in semiconductors under the action of electromagnetic fields and laser pulses, offering a detailed analysis of carrier behavior and wave transmission in rotating systems. Recent advancements in semiconductor thermoelastic analysis include the work by Salah et al.^19^, focusing on photothermal effects with magnetic fields and initial stress, and the study by Abo-Dahab et al.^20^, which incorporates electromagnetic and laser influences on excitation wave behavior. This work presents a comprehensive analysis of thermomechanical deformation in a nonlocal orthotropic medium subjected to simultaneous magnetic and rotational effects, employing the Green–Naghdi III model. The study aims to elucidate the complex interplay between these multifaceted influences, contributing to a deeper understanding of material response under coupled field conditions. In recent years, researchers have extensively focused on the wave’s propagation in a photothermal semiconducting medium (see for example^21–25^ and several references therein.

The present investigation is concerned with studying the two-dimensional disturbances in an infinite, homogeneous, orthotropic, rotating thermoelastic medium subjected to a magnetic field and governed by nonlocal theory within the framework of Green–Naghdi (G-N) theory of type III. A constant thermal load is applied on the boundary surface. Using normal mode analysis, the displacement components, stress distribution, and temperature field are derived. Numerical simulations, conducted for cobalt as the material model, explore the effects of time, rotation, nonlocality, and magnetic field on the system’s behavior. The effect of time, rotation, nonlocality, and magnetic field on the components of displacement, temperature distribution, and stress distribution has been shown graphically. The results highlight the significant influence of each parameter on the thermoelastic response. These findings have meaningful implications across various engineering disciplines, including soil dynamics, seismology, geophysics, and earthquake engineering.

## Formulation of the problem

**Fig. 1:**
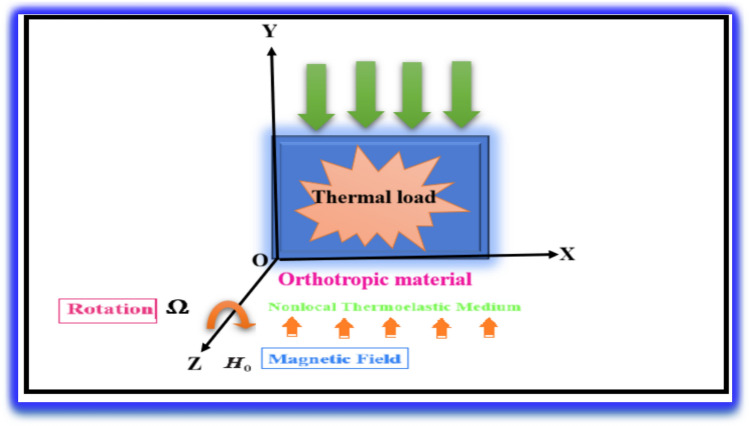
Schematic of the problem.

In the present mathematical model, we consider a rotating, homogeneous, orthotropic thermoelastic solid medium within the framework of the Green–Naghdi Type III (GN-III) model, taking into account the influences of a nonlocal parameter and an applied magnetic field. The coordinate system is established with its origin at point O, located on the horizontal surface, and the vertical axis extends downward into the solid half-space ($x, y\ge 0$), as illustrated in Fig. 1. The primary field variables are functions of the spatial coordinates *x*, *y*, and time *t*, and are assumed to be independent of the vertical coordinate. A thermal load is introduced on the surface within the *x–y* plane, which gradually diminishes as *x* tends toward infinity. The equation of motion for displacement in the rotating medium includes two additional terms due to rotation: the centripetal acceleration term $\vec{\Omega }^{ \wedge } \left( {\vec{\Omega }^{ \wedge } \vec{u}} \right)$, which arises from purely time-dependent motion, and the Coriolis acceleration term $\left( {2{\vec{\Omega }}^{ \wedge } \overrightarrow {{{\dot{\mathrm{u}}}}} } \right)$, which accounts for the motion in a rotating frame of reference. Here, Ω is the angular velocity vector, and the displacement components are defined as (*u* = *u (x, y, t)*) and (*v* = *v (x, y, t)*).

The equations of motion in the absence of the body forces taken from^13^, which is stated as follows:

1 $$\rho \left(1-{{L}_{N}}^{2}{\nabla }^{2}\right)\frac{{\partial }^{2}u}{\partial {t}^{2}}-{\rho\Omega }^{2}u+2\rho\Omega \frac{\partial v}{\partial t}=\left({c}_{11}+{\mu }_{e}{\rm H}_{^\circ }^{2}\right)\frac{{\partial }^{2}u}{\partial {x}^{2}}+{c}_{44}\frac{{\partial }^{2}u}{\partial {y}^{2}}+\left({c}_{12}+{c}_{44}+{\mu }_{e}{\rm H}_{^\circ }^{2}\right)\frac{{\partial }^{2}v}{\partial x\partial y}-{\beta }_{1}\frac{\partial T}{\partial x},$$

2 $$\rho \left(1-{{L}_{N}}^{2}{\nabla }^{2}\right)\frac{{\partial }^{2}v}{\partial {t}^{2}}-{\rho\Omega }^{2}v-2\rho\Omega \frac{\partial \mathrm{u}}{\partial \mathrm{t}}=\left({c}_{11}+{\mu }_{e}{\rm H}_{^\circ }^{2}\right)\frac{{\partial }^{2}v}{\partial {y}^{2}}+{c}_{44}\frac{{\partial }^{2}v}{\partial {x}^{2}}+\left({c}_{12}+{c}_{44}+{\mu }_{e}{\rm H}_{^\circ }^{2}\right)\frac{{\partial }^{2}u}{\partial x\partial y}-{\beta }_{2}\frac{\partial T}{\partial y},$$

The heat conduction equation is

3 $$\left( {K^{*} + K\frac{\partial }{{\partial t}}} \right)\left( {\frac{{\partial ^{2} T}}{{\partial x^{2} }} + \frac{{\partial ^{2} T}}{{\partial y^{2} }}} \right) = \frac{\partial }{{\partial t}}\left[ {\rho c_{e} \frac{{\partial {\mathrm{T}}}}{{\partial {\mathrm{t}}}} + T_{o} \frac{\partial }{{\partial t}}\left( {\beta _{1} \frac{{\partial {\mathrm{u}}}}{{\partial x}} + \beta _{2} \frac{{\partial {\mathrm{v}}}}{{\partial y}}} \right)} \right],$$

The stresses components are

4 $${\sigma }_{xx}={c}_{11}\frac{\partial u}{\partial x}+{c}_{12}\frac{\partial v}{\partial y}-{\beta }_{1}\left(T-{T}_{o}\right),$$

5 $${\sigma }_{yy}={c}_{12}\frac{\partial u}{\partial x}+{c}_{22}\frac{\partial v}{\partial y}-{\beta }_{2}\left(T-{T}_{o}\right),$$

6 $${\sigma }_{xy}={c}_{44}\left(\frac{\partial u}{\partial y}+\frac{\partial v}{\partial x}\right).$$

The dimensionless variables listed below can be used to improve the transformation of the main quantities in the dimensionless case:

7 $$\left( {{\text{ }}x^{\prime},{\text{ }}y^{\prime},{\text{ }}u^{\prime},{\text{ }}v^{\prime}} \right) = c_{o} \eta \left( {x,y,u,v} \right),\sigma ^{\prime}_{{ij}} = \frac{1}{{c_{{11}} }}\sigma _{{ij}} ,T^{\prime } = \frac{{T - T_{o} }}{{T_{o} }},t^{\prime } = \eta t,\Omega ^{\prime} = \frac{\Omega }{\eta },c_{o}^{2} = \frac{{c_{{11}} }}{\rho },\eta = \frac{{\rho c_{e} }}{K}$$

To simplify the presentation of the governing Eqs. (1)– (6) with Eq. (7) dashes—are removed. Consequently, it generates

8 $$\left(1-{a}_{o}{\nabla }^{2}\right)\frac{{\partial }^{2}u}{\partial {t}^{2}}-{\Omega }^{2}u+2\Omega \frac{\partial v}{\partial t}={a}_{1}\frac{{\partial }^{2}u}{\partial {x}^{2}}+{a}_{2}\frac{{\partial }^{2}u}{\partial {y}^{2}}+ {a}_{3}\frac{{\partial }^{2}v}{\partial x\partial y}-{a}_{4}\frac{\partial T}{\partial x},$$

9 $$\left(1-{a}_{o}{\nabla }^{2}\right)\frac{{\partial }^{2}v}{\partial {t}^{2}}-{\Omega }^{2}v-2\Omega \frac{\partial \mathrm{u}}{\partial \mathrm{t}}={a}_{1}\frac{{\partial }^{2}v}{\partial {y}^{2}}+{a}_{2}\frac{{\partial }^{2}v}{\partial {x}^{2}} +{a}_{3}\frac{{\partial }^{2}u}{\partial x\partial y}-{a}_{5}\frac{\partial T}{\partial y},$$

10 $$\left( {K^{*} + K\eta \frac{\partial }{{\partial t}}} \right)\left( {\frac{{\partial ^{2} T}}{{\partial x^{2} }} + \frac{{\partial ^{2} T}}{{\partial y^{2} }}} \right) = \frac{\partial }{{\partial t}}\left[ {a_{6} \frac{{\partial {\mathrm{T}}}}{{\partial {\mathrm{t}}}} + \frac{\partial }{{\partial t}}\left( {a_{7} \frac{{\partial {\mathrm{u}}}}{{\partial x}} + a_{8} \frac{{\partial {\mathrm{v}}}}{{\partial y}}} \right)} \right],$$

11 $${\sigma }_{xx}=\frac{\partial u}{\partial x}+{a}_{9}\frac{\partial v}{\partial y}-{a}_{10}T,$$

12 $${\sigma }_{yy}={a}_{9}\frac{\partial u}{\partial x}+{a}_{11}\frac{\partial v}{\partial y}-{a}_{12}T,$$

13 $${\sigma }_{xy}={a}_{13}\left(\frac{\partial u}{\partial y}+\frac{\partial v}{\partial x}\right).$$

where,

$$\begin{gathered} a_{o} = c_{o}^{2} \eta ^{2} L_{N}^{2} ,\;a_{1} = \frac{{~c_{o}^{2} \left( {c_{{11}} + \mu _{e} {\rm H}_{^\circ }^{2} } \right)}}{\rho },\;a_{2} = \frac{{c_{o}^{2} c_{{44}} }}{\rho },\,a_{3} = \frac{{c_{o}^{2} \left( {~c_{{12}} + c_{{44}} + \mu _{e} {\rm H}_{^\circ }^{2} } \right)}}{\rho }, \hfill \\ a_{4} = \frac{{~c_{o}^{2} T_{o} \beta _{1} }}{\rho },\;a_{5} = \frac{{~c_{o}^{2} T_{o} \beta _{2} }}{\rho },\;a_{6} = \frac{{\rho c_{e} }}{{c_{o}^{2} }},\;a_{7} = \frac{{\beta _{1} }}{{c_{o}^{2} }},\;a_{8} = \frac{{\beta _{2} }}{{c_{o}^{2} }},\;a_{9} = \frac{{~c_{{12}} }}{{c_{{11}} }}, \hfill \\ a_{{10}} = \frac{{T_{o} \beta _{1} }}{{c_{{11}} }},\;a_{{11}} = \frac{{~c_{{22}} }}{{c_{{11}} }},\;a_{{12}} = \frac{{T_{o} \beta _{2} }}{{c_{{11}} }},\;a_{{13}} = \frac{{~c_{{44}} }}{{c_{{11}} }}. \hfill \\ \end{gathered}$$

## Solution of the problem

 This section, normal mode technique is employed, which has the advantage of finding the exact solutions without any assumed constraints on the field variables. So, the physical variables under consideration can be decomposed in terms of normal modes^26^ in the following form

14 $$\left(u,v,T,{\sigma }_{ij}\right)\left(x,y,t\right)=\left({u}^{*},{v}^{*},{T}^{*},{{\sigma }_{ij}}^{*}\right)\left(x\right){e}^{\left(\omega t+iby\right)}.$$

where $\omega , i, and b$ refer to the angular frequency, the imaginary number and the wave number in the y- direction. Applying Eq. (14) in Eqs. (8–10), we have

15 $$\frac{{d}^{2}{u}^{*}}{d{x}^{2}}={a}_{14}{u}^{*}+{a}_{15}{v}^{*}-{a}_{16}\frac{d{v}^{*}}{dx}+{a}_{17}\frac{d{T}^{*}}{dx},$$

16 $$\frac{{d}^{2}{v}^{*}}{d{x}^{2}}={a}_{18}{u}^{*}+{a}_{19}{v}^{*}+{a}_{20}{T}^{*}-{a}_{21}\frac{d{u}^{*}}{dx},$$

17 $$\frac{{d}^{2}{T}^{*}}{d{x}^{2}}={a}_{22}{v}^{*}+{a}_{23}{T}^{*}+{a}_{24}\frac{d{u}^{*}}{dx}$$

where,

$$\begin{gathered} a_{{14}} = \frac{{\omega ^{2} + a_{o} \omega ^{2} b^{2} + a_{2} b^{2} - \Omega ^{2} }}{{a_{o} \omega ^{2} + a_{1} }},\;a_{{15}} = \frac{{2\omega \Omega }}{{a_{o} \omega ^{2} + a_{1} }},\;a_{{16}} = \frac{{iba_{3} }}{{a_{o} \omega ^{2} + a_{1} }}, \hfill \\ a_{{17}} = \frac{{a_{4} }}{{a_{o} \omega ^{2} + a_{1} }},a_{{18}} = \frac{{ - 2\omega \Omega }}{{a_{o} \omega ^{2} + a_{2} }},\;a_{{19}} = \frac{{\omega ^{2} + a_{o} \omega ^{2} b^{2} + a_{1} b^{2} - \Omega ^{2} }}{{a_{o} \omega ^{2} + a_{2} }}, \hfill \\ a_{{20}} = \frac{{iba_{5} }}{{a_{o} \omega ^{2} + a_{2} }},\;a_{{21}} = \frac{{iba_{3} }}{{a_{o} \omega ^{2} + a_{2} }},a_{{22}} = \frac{{iba_{8} \omega ^{2} }}{{K^{*} + K\eta \omega }},\;a_{{23}} = \frac{{a_{6} \omega ^{2} + b^{2} }}{{K^{*} + K\eta \omega }},\;a_{{24}} = \frac{{a_{7} \omega ^{2} }}{{K^{*} + K\eta \omega }}. \hfill \\ \end{gathered}$$

Equations (15), (16) and (17) can be expressed as a differential equation with a vector–matrix as below:

18 $$\frac{d\varrho }{dx}=A\varrho .$$

where,

19 $$\varrho =\left(\begin{array}{c}{u}^{*}\\ {v}^{*}\\ {T}^{*}\\ \frac{d{u}^{*}}{dx}\\ \frac{d{v}^{*}}{dx}\\ \frac{d{T}^{*}}{dx}\end{array}\right),A=\left(\begin{array}{cccccc}0& 0& 0& 1& 0& 0\\ 0& 0& 0& 0& 1& 0\\ 0& 0& 0& 0& 0& 1\\ {a}_{14}& {a}_{15}& 0& 0& -{a}_{16}& {a}_{17}\\ {a}_{18}& {a}_{19}& {a}_{20}& -{a}_{21}& 0& 0\\ 0& {a}_{22}& {a}_{23}& {a}_{24}& 0& 0\end{array}\right)$$

As in Das and Bhakta^26^, we now use the eigenvalue technique to solve Eq. (18) a matrix A’s characteristic equation, which has the form

20 $${\lambda }^{6}+{m}_{11}{\lambda }^{4}+{m}_{22}{\lambda }^{2}+{m}_{33}=0.$$

where,

$$\begin{gathered} m_{11} = - a_{14} - a_{19} - a_{16} a_{21} - a_{23} - a_{17} a_{24} , \hfill \\ m_{22} = - a_{15} a_{18} + a_{14} a_{19} - a_{20} a_{22} + a_{17} a_{21} a_{22} \hfill \\ \quad \quad \quad + a_{14} a_{23} + a_{19} a_{23} + a_{16} a_{21} a_{23} ,\; + a_{17} a_{19} a_{24} + a_{16} a_{20} a_{24} \; \hfill \\ m_{33} = a_{14} a_{20} a_{22} + a_{15} a_{18} a_{23} - a_{14} a_{19} a_{23} . \hfill \\ \end{gathered}$$

The roots of the Eq. (20) are ${\lambda }_{i}=\pm {\lambda }_{1},\pm {\lambda }_{2},\pm {\lambda }_{3},i=\mathrm{1,2},3$, which are stated in the appendix A in the end of the paper.

The right eigenvector.

$\chi ={\left[{\chi }_{1},{\chi }_{2},{\chi }_{3},{\chi }_{4},{\chi }_{5},{\chi }_{6}\right]}^{T}$ corresponding to eigenvalue λ can be considered as

21 $$\chi = \left( {\begin{array}{*{20}c} {\frac{{a_{{17}} \left( {a_{{16}} \lambda - a_{{15}} } \right)\left( {a_{{18}} - a_{{21}} \lambda } \right) + a_{{17}} \left( {a_{{21}} \lambda - a_{{18}} } \right)\left( {a_{{16}} \lambda - a_{{15}} } \right)}}{{(a_{{14}} - \lambda ^{2} )}} - a_{{17}} \left( {a_{{19}} - \lambda ^{2} } \right)} \\ {a_{{17}} a_{{18}} - a_{{17}} a_{{21}} \lambda } \\ {\frac{1}{\lambda }\left[ {\left( {a_{{19}} - \lambda ^{2} } \right)\left( {a_{{14}} - \lambda ^{2} } \right) - \left( {a_{{21}} \lambda - a_{{18}} } \right)\left( {a_{{16}} \lambda - a_{{15}} } \right)} \right]} \\ {\frac{{a_{{17}} \lambda \left( {a_{{16}} \lambda - a_{{15}} } \right)\left( {a_{{18}} - a_{{21}} \lambda } \right) + a_{{17}} \lambda \left( {a_{{21}} \lambda - a_{{18}} } \right)\left( {a_{{16}} \lambda - a_{{15}} } \right)}}{{(a_{{14}} - \lambda ^{2} )}} - a_{{17}} \lambda \left( {a_{{19}} - \lambda ^{2} } \right)} \\ {a_{{17}} a_{{18}} \lambda - a_{{17}} a_{{21}} \lambda ^{2} } \\ {\left( {a_{{19}} - \lambda ^{2} } \right)\left( {a_{{14}} - \lambda ^{2} } \right) - \left( {a_{{21}} \lambda - a_{{18}} } \right)\left( {a_{{16}} \lambda - a_{{15}} } \right)} \\ \end{array} } \right)$$

It is simple to determine the eigenvector $v$ that corresponds to the eigenvalue from Eq. (21). The notations used in the remainder of the work are as follows:

$${\chi }_{1}={\left[\chi \right]}_{\lambda ={\lambda }_{1}}{\chi }_{2}={\left[\chi \right]}_{\lambda ={\lambda }_{2}}{\chi }_{3}={\left[\chi \right]}_{\lambda ={\lambda }_{3}}{\chi }_{4}={\left[\chi \right]}_{\lambda ={\lambda }_{4}}{\chi }_{5}={\left[\chi \right]}_{\lambda ={\lambda }_{5}}{\chi }_{6}={\left[\chi \right]}_{\lambda ={\lambda }_{6}}$$

Considering the regularity criterion at infinity, the answer to Eq. (18) is as follows:

22 $${\varrho } = \sum\nolimits_{{i = 1}}^{3} {A_{i} } \chi _{i} e^{{ - \lambda _{i} x}} = A_{1} \chi _{1} e^{{ - \lambda _{1} x}} + A_{2} \chi _{2} e^{{ - \lambda _{2} x}} + A_{3} \chi _{3} e^{{ - \lambda _{3} x}} ,\left( {x \ge 0} \right).$$

where ${A}_{1},{A}_{2},{A}_{3}$ are constants that the problem’s boundary conditions dictate. Based on (19), (20) and (22), we have

23 $${T}^{*}\left(x\right)={A}_{1}{\Gamma }_{1}{e}^{{-\lambda }_{1}x}+{A}_{2}{\Gamma }_{2}{e}^{-{\lambda }_{2}x}+{A}_{3}{\Gamma }_{3}{e}^{-{\lambda }_{3}x},$$

24 $${u}^{*}\left(x\right)={A}_{1}{\Pi }_{1}{e}^{{-\lambda }_{1}x}+{A}_{2}{\Pi }_{2}{e}^{{-\lambda }_{2}x}+{A}_{3}{\Pi }_{3}{e}^{{-\lambda }_{3}x},$$

25 $${v}^{*}\left(x\right)={A}_{1}{\Delta }_{1}{e}^{{-\lambda }_{1}x}+{A}_{2}{\Delta }_{2}{e}^{-{\lambda }_{2}x}+{A}_{3}{\Delta }_{3}{e}^{-{\lambda }_{3}x},$$

26 $${{\sigma }_{xx}}^{*}\left(x\right)={A}_{1}{\pi }_{1}{e}^{{-\lambda }_{1}x}+{A}_{2}{\pi }_{2}{e}^{-{\lambda }_{2}x}+{A}_{3}{\pi }_{3}{e}^{-{\lambda }_{3}x},$$

27 $${{\sigma }_{yy}}^{*}\left(x\right)={A}_{1}{\upsigma }_{1}{e}^{{-\lambda }_{1}x}+{A}_{2}{\upsigma }_{2}{e}^{-{\lambda }_{2}x}+{A}_{3}{\upsigma }_{3}{e}^{-{\lambda }_{3}x},$$

28 $$\sigma_{xy}^{*} \left( x \right) = A_{1} \alpha_{1} e^{{ - \lambda_{1} x}} + A_{2} \alpha_{2} e^{{ - \lambda_{2} x}} + A_{3} \alpha_{3} e^{{ - \lambda_{3} x}} .$$

where,

$$\begin{gathered} \Gamma _{1} = \frac{1}{{\lambda _{1} }}\left[ {\left( {a_{{19}} - \lambda _{1} ^{2} } \right)\left( {a_{{14}} - \lambda _{1} ^{2} } \right) - \left( {a_{{21}} \lambda _{1} - a_{{18}} } \right)\left( {a_{{16}} \lambda _{1} - a_{{15}} } \right)} \right], \hfill \\ \Gamma _{2} = \frac{1}{{\lambda _{2} }}\left[ {\left( {a_{{19}} - \lambda _{2} ^{2} } \right)\left( {a_{{14}} - \lambda _{2} ^{2} } \right) - \left( {a_{{21}} \lambda _{2} - a_{{18}} } \right)\left( {a_{{16}} \lambda _{2} - a_{{15}} } \right)} \right], \hfill \\ \Gamma _{3} = \frac{1}{{\lambda _{3} }}\left[ {\left( {a_{{19}} - \lambda _{3} ^{2} } \right)\left( {a_{{14}} - \lambda _{3} ^{2} } \right) - \left( {a_{{21}} \lambda _{3} - a_{{18}} } \right)\left( {a_{{16}} \lambda _{3} - a_{{15}} } \right)} \right], \hfill \\ \end{gathered}$$

$$\begin{gathered} \Pi _{1} = \frac{{a_{{17}} \left( {a_{{16}} \lambda _{1} - a_{{15}} } \right)\left( {a_{{18}} - a_{{21}} \lambda _{1} } \right) + a_{{17}} \left( {a_{{21}} \lambda _{1} - a_{{18}} } \right)\left( {a_{{16}} \lambda _{1} - a_{{15}} } \right)}}{{(a_{{14}} - \lambda _{1} ^{2} )}} - a_{{17}} \left( {a_{{19}} - \lambda _{1} ^{2} } \right), \hfill \\ \Pi _{2} = \frac{{a_{{17}} \left( {a_{{16}} \lambda _{2} - a_{{15}} } \right)\left( {a_{{18}} - a_{{21}} \lambda _{2} } \right) + a_{{17}} \left( {a_{{21}} \lambda _{2} - a_{{18}} } \right)\left( {a_{{16}} \lambda _{2} - a_{{15}} } \right)}}{{(a_{{14}} - \lambda _{2} ^{2} )}} - a_{{17}} \left( {a_{{19}} - \lambda _{2} ^{2} } \right), \hfill \\ \Pi _{3} = \frac{{a_{{17}} \left( {a_{{16}} \lambda _{3} - a_{{15}} } \right)\left( {a_{{18}} - a_{{21}} \lambda _{3} } \right) + a_{{17}} \left( {a_{{21}} \lambda _{3} - a_{{18}} } \right)\left( {a_{{16}} \lambda _{3} - a_{{15}} } \right)}}{{(a_{{14}} - \lambda _{3} ^{2} )}} - a_{{17}} \left( {a_{{19}} - \lambda _{3} ^{2} } \right), \hfill \\ \end{gathered}$$

$$\Delta _{1} = a_{{17}} a_{{18}} - a_{{17}} a_{{21}} \lambda _{1} ,\;\Delta _{2} = a_{{17}} a_{{18}} - a_{{17}} a_{{21}} ,\;\Delta _{3} = a_{{17}} a_{{18}} - a_{{17}} a_{{21}} \lambda _{3} ,$$

$$\pi _{1} = - \lambda _{1} \Pi _{1} + iba_{9} \Delta _{1} - a_{{10}} \Gamma _{1} ,\;\pi _{2} = - \lambda _{2} \Pi _{2} + iba_{9} \Delta _{2} - a_{{10}} \Gamma _{2} ,\;\pi _{3} = - \lambda _{3} \Pi _{3} + iba_{9} \Delta _{3} - a_{{10}} \Gamma _{3} ,$$

$$\sigma _{1} = - a_{9} \lambda _{1} \Pi _{1} + iba_{{11}} \Delta _{1} - a_{{12}} \Gamma _{1} ,\;\sigma _{2} = - a_{9} \lambda _{2} \Pi _{2} + iba_{{11}} \Delta _{2} - a_{{12}} \Gamma _{2} ,\;\sigma _{3} = - a_{9} \lambda _{3} \Pi _{3} + iba_{{11}} \Delta _{3} - a_{{12}} \Gamma _{3} ,$$

$$\alpha_{1} = iba_{13} \Pi_{1} - a_{13} \lambda_{1} \Delta_{1} \alpha_{2} = iba_{13} \Pi_{2} - a_{13} \lambda_{2} \Delta_{2} \alpha_{3} = iba_{13} \Pi_{3} - a_{13} \lambda_{3} \Delta_{3} .$$

## Boundary conditions

In this part, we will apply these boundary conditions to our problem. We assume that the free surface in our suggested model is traction-free.

In the present formulation, appropriate mechanical and thermal boundary conditions are prescribed to ensure a physically meaningful and reproducible problem definition. The material is assumed to be homogeneous, orthotropic, and thermally conducting, and the boundary $x=0$ represents a free surface subjected to a dynamic thermal load.

### Mechanical boundary conditions

The free surface is assumed to be traction-free, implying that no external mechanical forces act on the boundary. Consequently, both the normal and shear stress components vanish at $x=0$:

29 $$\sigma _{{xx}} ^{*} = 0,\;\;x = 0,$$

30 $$\sigma _{{xy}} ^{*} = 0,\;\;x = 0.$$

These conditions correspond to a mechanically traction-free boundary conditions on the surface, which is a standard assumption for thermoelastic wave-propagation problems. They also ensure that any deformation arises solely from the thermal load and the nonlocal thermoelastic coupling, rather than from externally applied mechanical tractions.

### Thermal boundary condition

The boundary is subjected to a time-harmonic thermal load of the form where *P* = ${P}^{*}{e}^{(\omega t+iby)}$ denotes the amplitude (intensity) of the imposed heat flux,

ω is the excitation frequency, and y represents the direction transverse to heat propagation. Accordingly, the thermal boundary condition is expressed as:

31 $${T}^{*}=P\;\;\;\;\text{at }x=0,$$

which it implies that the surface temperature is directly driven by the applied oscillatory thermal input. This condition establishes a well-defined thermal disturbance that enables consistent comparison with classical local thermoelastic models in later sections.

For the constants ${A}_{1}$, ${A}_{2}$ and ${A}_{3}$, we obtain three equations by combining Eqs. (29–31) with (23), (26) and (28)

32 $$\begin{gathered} \pi _{1} A_{1} + \pi _{2} A_{2} + \pi _{3} A_{3} = 0, \hfill \\ \alpha _{1} A_{1} + \alpha _{2} A_{2} + \alpha _{3} A_{3} = 0, \hfill \\ A_{1} \Gamma _{1} + A_{2} \Gamma _{2} + A_{3} \Gamma _{3} = P. \hfill \\ \end{gathered}$$

To calculate the constants ${A}_{1}$, ${A}_{2}$, and ${A}_{3}$ Cramer’s method is applied on Eqs. (32).

33 $${A}_{1}=\frac{\Delta {A}_{1}}{\Delta }, {A}_{2}=\frac{\Delta {A}_{2}}{\Delta }, {A}_{3}=\frac{\Delta {A}_{3}}{\Delta }.$$

Using Eqs. (14) and (23–28), one may calculate the dimensionless temperature *T*, displacements *u* and *v*, and stress components ${\sigma }_{xx},{\sigma }_{yy},$ and ${\sigma }_{xy}$.

34 $$T\left(x,y,t\right)=\left\{\sum_{i=1}^{3}{A}_{i}{\Gamma }_{i}{e}^{-{\lambda }_{i}x}\right\}{e}^{\left(\omega t+iey\right)},$$

35 $$u\left(x,y,t\right)=\left\{\sum_{i=1}^{3}{A}_{i}{{\Pi }_{i}e}^{-{\lambda }_{i}x}\right\}{e}^{\left(\omega t+iey\right)},$$

36 $$v\left(x,y,t\right)=\left\{\sum_{i=1}^{3}{A}_{i}{\Delta }_{i}{e}^{{-\lambda }_{i}x}\right\}{e}^{\left(\omega t+iey\right)},$$

37 $${\sigma }_{xx}\left(x,y,t\right)=\left\{\sum_{i=1}^{3}{A}_{i}{\pi }_{i}{e}^{{-\lambda }_{i}x}\right\}{e}^{\left(\omega t+iey\right)},$$

38 $${\sigma }_{yy}\left(x,y,t\right)=\left\{\sum_{i=1}^{3}{A}_{i}{\upsigma }_{i}{e}^{{-\lambda }_{i}x}\right\}{e}^{\left(\omega t+iey\right)},$$

39 $$\sigma_{xy} \left( {x,y,t} \right) = \left\{ {\mathop \sum \limits_{i = 1}^{3} A_{i} \alpha_{i} e^{{ - \lambda_{i} x}} } \right\}e^{{\left( {\omega t + iey} \right)}} .$$

## Numerical results and discussion

This section presents the numerical simulation of an orthotropic magneto thermoelastic half-space incorporating nonlocal effects, as well as the influence of rotation. The analysis will focus on the non-local model in both space and time, which incorporates rotation and magnetic field, exploring how these factors influence the behavior of various physical quantities. The simulations are performed using MATHEMATICA software to evaluate key physical fields, including temperature, displacement components, shear stress, and normal stresses. The material properties and parameters required for the model are taken from Biswas and Lataa^27,28^, with the physical constants of cobalt expressed in SI units.

$$\begin{gathered} c_{{11}} = 3.071 \times 10^{{11}} {\text{ Kg}}.{\mathrm{m}}^{{ - 1}} .{\mathrm{s}}^{{ - 2}} ,c_{{12}} = 1.650 \times 10^{{11}} {\text{ Kg}}.{\mathrm{m}}^{{ - 1}} .{\mathrm{s}}^{{ - 2}} ,Ce = 427{\text{ J}}/{\text{Kg K}}, \hfill \\ c_{{22}} = 3.581 \times 10^{{11}} {\text{ Kg}}.{\mathrm{m}}^{{ - 1}} .{\mathrm{s}}^{{ - 2}} ,c_{{44}} = 1.510 \times 10^{{11}} {\text{ Kg}}.{\mathrm{m}}^{{ - 1}} .{\mathrm{s}}^{{ - 2}} ,\rho = 8836{\text{ Kg}}.{\mathrm{m}}^{{ - 3}} , \hfill \\ T_{o} = 293{\text{ K}},\mu _{e} = 4\pi \times 10^{{ - 7}} \frac{{\mathrm{H}}}{{\mathrm{m}}},{\mathrm{K}} = 3.86 \times 102{\text{ W s}}^{{ - 1}} ,K^{*} = 1.54 \times 102{\text{ W s}}^{{ - 1}} , \hfill \\ \beta _{1} = 7.04 \times 10^{{ - 5}} {\text{ Nm}}^{2} {\mathrm{k}}^{{ - 1}} \beta _{2} = 6.90 \times 10^{{ - 5}} {\text{ Nm}}^{2} {\mathrm{k}}^{{ - 1}} t = 0.1{\mathrm{s}}. \hfill \\ \end{gathered}$$

**Fig. 2:**
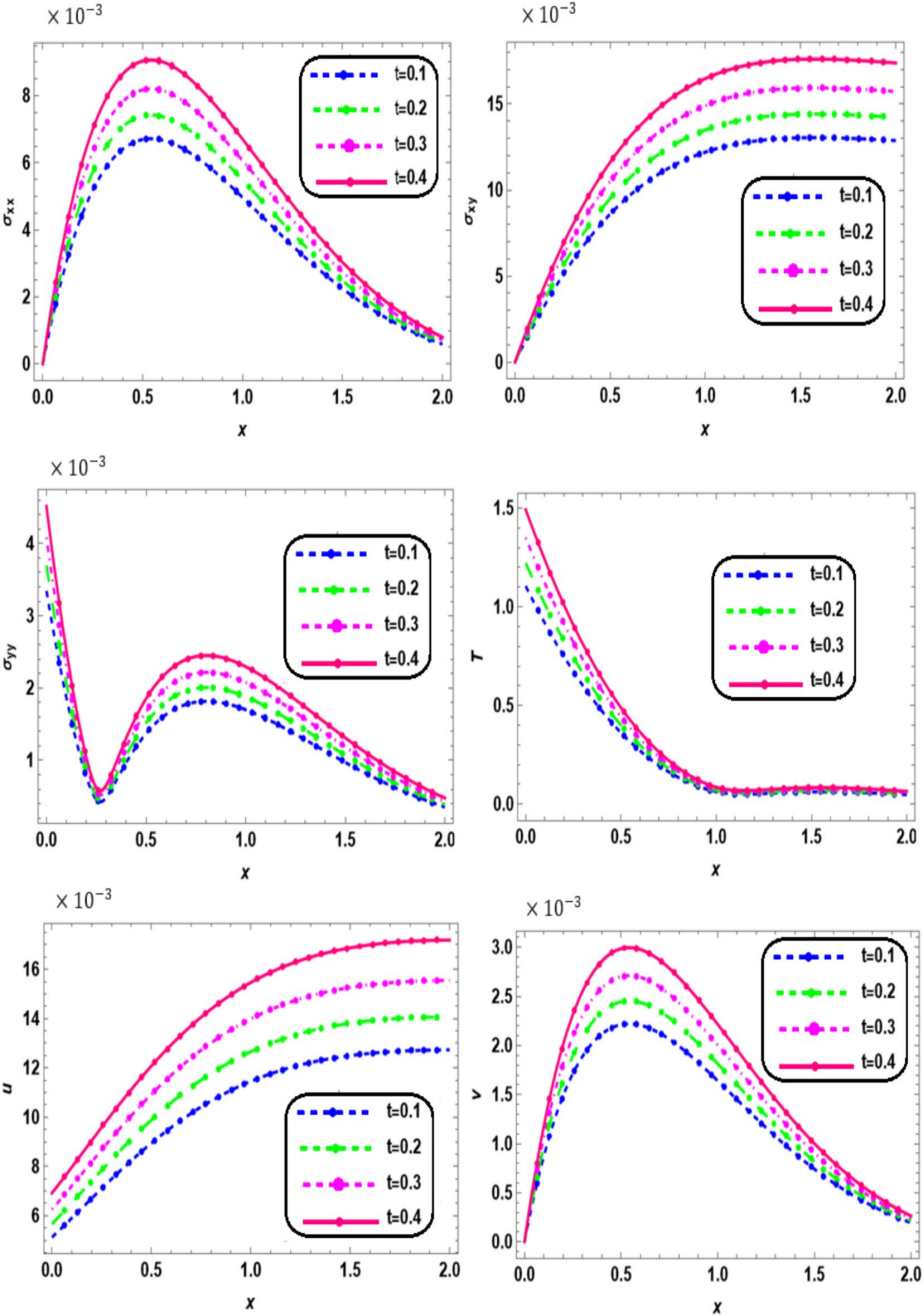
Effect of time on the main physical quantities versus* x*.

**Fig. 3:**
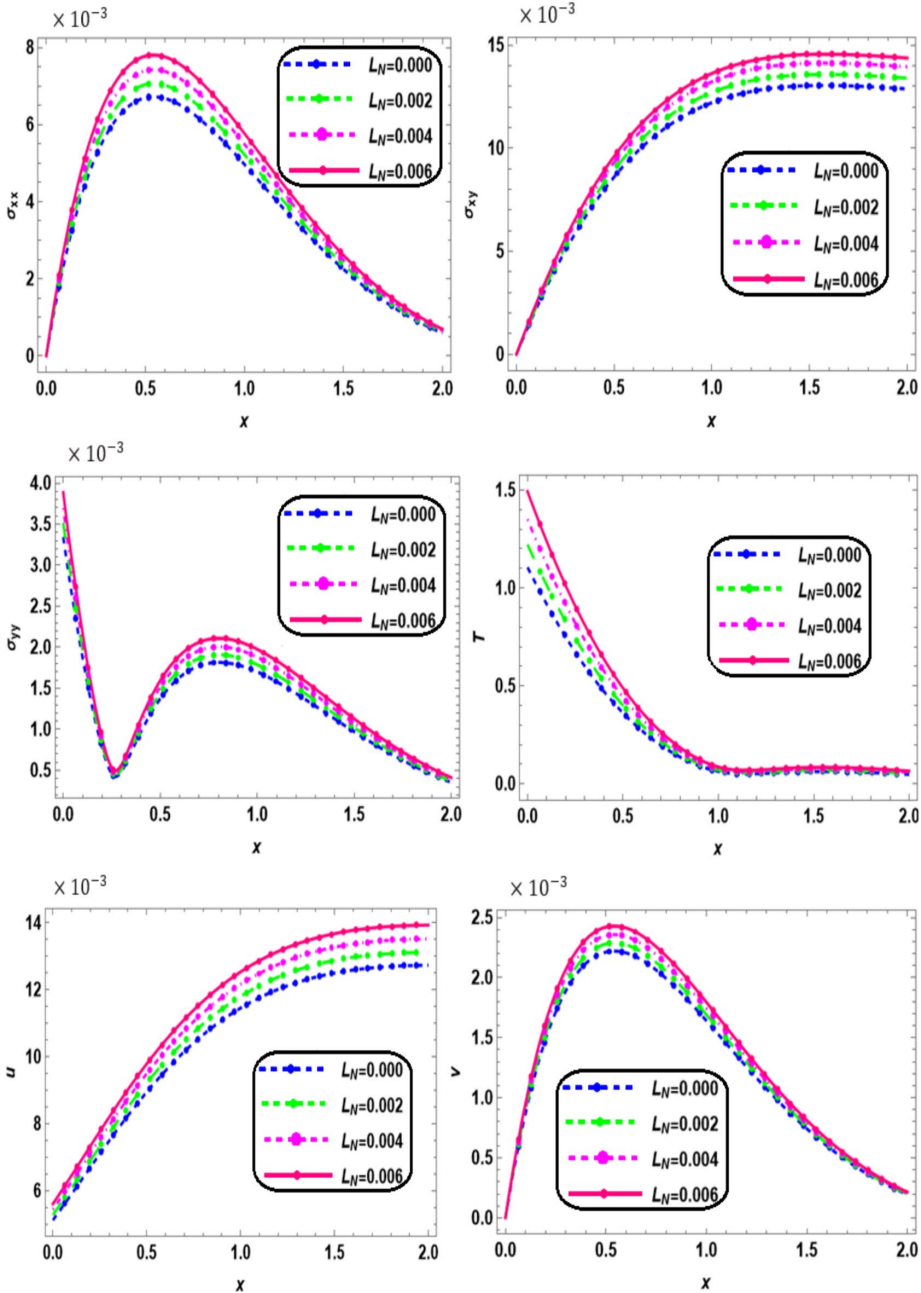
Effect of nonlocal on the main physical quantities versus* x*.

**Fig. 4:**
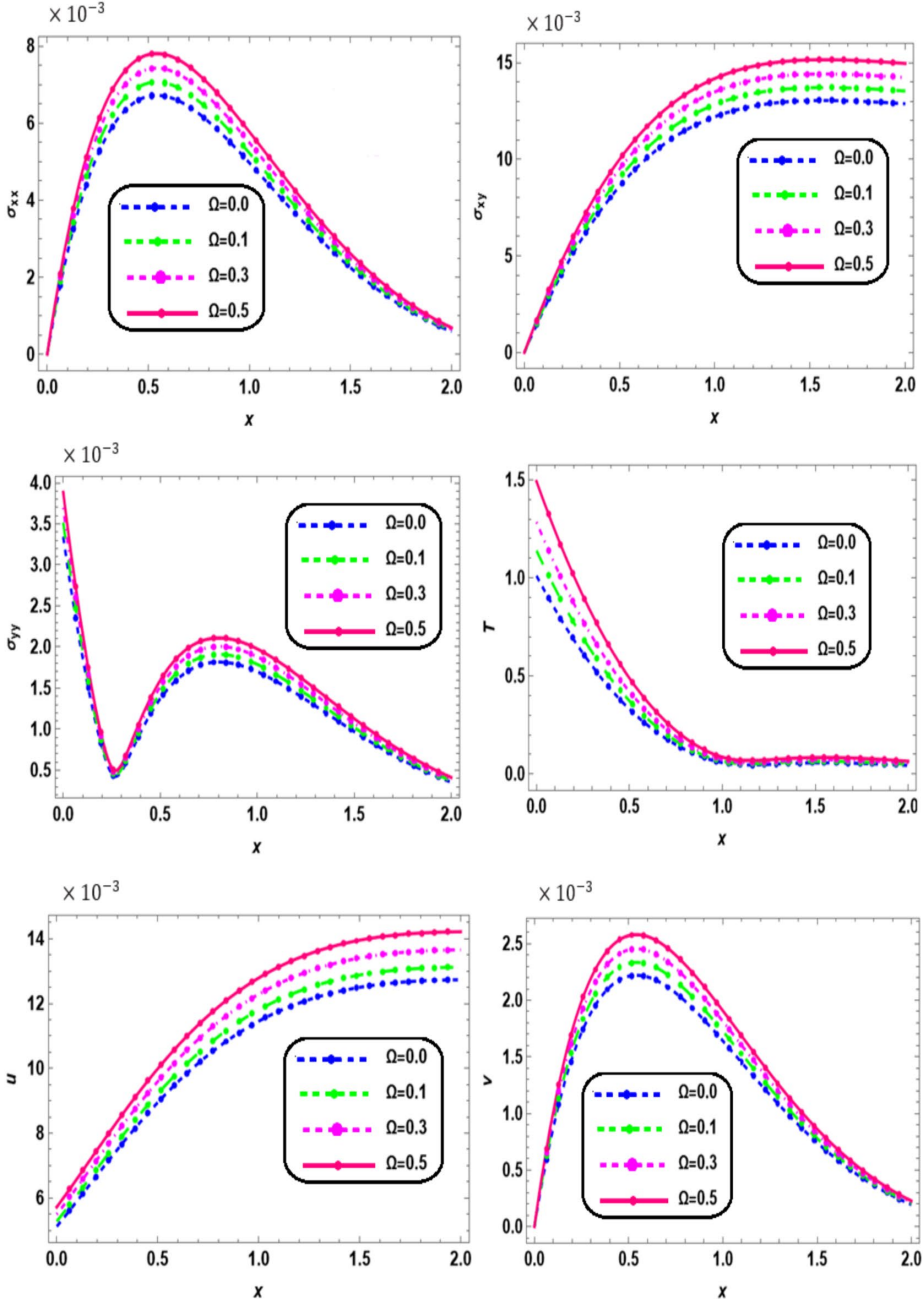
Effect of rotation on the physical quantities with *x*.

**Fig. 5:**
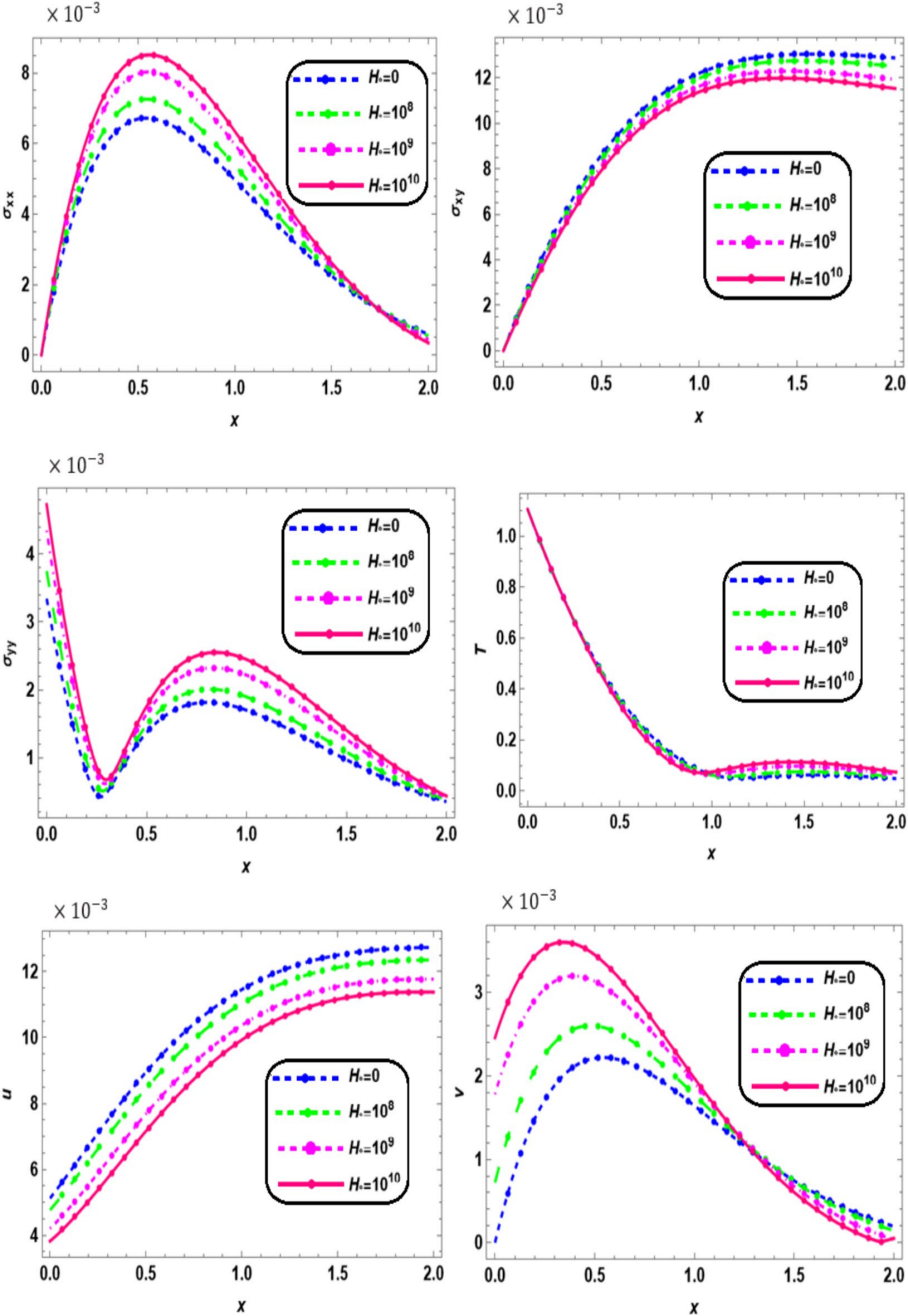
Effect of magnetic field on the physical quantities.

In Figs. 2, 3, 4 and 5, variation in the real component of the dimensionless field variables is plotted against the $x -$ axis on the plane $y = 0.5$ and at wave number $b=0.5.$

Figure 2 illustrates the variation of thermal stresses (${\sigma }_{xx}$, ${\sigma }_{xy}$, and ${\sigma }_{yy}$), displacement components (*u*, *v*), and temperature (*T*) along the *x-*axis for different values of time $t$ (0.1, 0.2, 0.3, and 0.4) for a nonlocal orthotropic rotating thermoelastic medium subjected to both a magnetic field and a thermal load. It is observed that all physical quantities increase with time within the interval 0 ≤ *x* ≤ 2. These results highlight the significant influence of time on stress distribution, displacement, and temperature evolution. The observed oscillatory behavior reflects the complex interaction between mechanical and thermal effects, emphasizing the need for time-dependent analysis when assessing material behavior under thermal loading. Understanding these transient responses is crucial for designing materials and structures capable of withstanding thermal variations, thereby ensuring long-term stability and performance. Initially, the amplitude experiences an exponential increase, followed by a subsequent increase, ultimately normal stress and shear stress approaching zero at initial distance $x$ . It is noticed that the solution curves for all four values of time follow the same pattern of variations of the temperature and converge to zero with an increase in distance $x$. These results obey the physical properties of thermoelasticity GN-III theory. This result is in a good agreement with the results obtained by Othman et al.^29^.

Figure 3 presents the variations in thermal stresses (${\sigma }_{xx},$ ${\sigma }_{xy},$ and ${\sigma }_{yy}$), displacements (*u*, *v*), and temperature (*T*) along the x-axis for different values of the nonlocal parameter $L_{N}$ (0, 0.002, 0.004, and 0.006) in a nonlocal orthotropic rotating thermoelastic medium subjected to both a magnetic field and thermal load. Within the interval 0 ≤ *x* ≤ 2, all physical quantities show an increasing trend with higher nonlocality. These findings highlight the significant impact of nonlocal effects on the mechanical and thermal responses of the material. The observed fluctuations in stress, displacement, and thermal diffusion underscore the complex interplay between nonlocal elasticity and material behavior under thermal conditions. A deeper understanding of these interactions is essential for the design of materials and structures where nonlocal effects are prominent, such as in nanoscale systems, porous media, and advanced composite materials. It is noticed that the non-local effects significantly affect the physical quantities variation behavior compared to non-local elastic materials. This result is in good agreement with the results obtained by Khader et al.^30^.

Figure 4 depicts the variations in thermal stresses (${\sigma }_{xx,}$ ${\sigma }_{xy},$ and ${\sigma }_{yy}$), displacements (*u*, *v*), and temperature (*T*) along the *x*-axis for different values of rotation $\Omega$ (0, 0.1, 0.3, and 0.5) in a nonlocal orthotropic rotating thermoelastic medium subjected to both a magnetic field and thermal load. Within the interval 0 ≤ *x* ≤ 2, all physical quantities are observed to increase with rotation. These results underscore the pronounced impact of rotation on stress distribution, displacement, and temperature evolution. They offer valuable insight into the coupled mechanical and thermal behavior of the system, highlighting the role of rotation and nonlocal effects in influencing stress redistribution, displacement variation, and thermal diffusion. Such understanding is essential for optimizing material performance in applications involving rotational dynamics and nonlocal interactions. It can be noted that rotation has a great expressive effect on the solutions of stress, displacements and temperature field. It is clearly observed that the mechanical waves are highly sensitive towards the characteristic rotation. The normal stress and shear distribution reveals how uncertainties in thermal and mechanical excitation influence stress wave propagation, which is particularly important in high-precision applications. This result is in good agreement with the results obtained by Bayones and Abd-Alla^31^.

Figure 5 shows the variations in thermal stresses (${\sigma }_{xx},$ ${\sigma }_{xy},$ and ${\sigma }_{yy}$), displacements (*u*, *v*), and temperature (*T*) along the x-axis for different values of magnetic field strengths (${H}_{o}$ = 0, ${10}^{8}$, ${10}^{9}$, and ${10}^{10}$) in a nonlocal orthotropic rotating thermoelastic medium subjected to both magnetic and thermal loading. Within the interval 0 ≤ *x* ≤ 2, the temperature (*T*), normal stresses (${\sigma }_{xx}$ and ${\sigma }_{yy}$), and vertical displacement (*v*) are observed to increase with increasing magnetic field strength. In contrast, the shear stress (${\sigma }_{xy}$) and horizontal displacement (*u*) also increase with ${H}_{o}$, though their variation is more sensitive to the magneto-mechanical coupling effects. These results indicate that the magnetic field intensifies the overall mechanical and thermal response of the material. All the curves in each figure have coincident initial coordinates (0,0). These facts agree well with the boundary conditions. Here we notice that values of $\sigma_{xx}$ and $\sigma_{xy}$ are large in the presence of a magnetic field in comparison to its absence, which shows that the magnetic field has an increasing effect on the profile of thermal stress. The increase in temperature and normal stresses with ${H}_{o}$ suggests enhanced resistance to thermal expansion and stronger internal forces due to magneto-thermoelastic coupling. The rise in displacements (*u* and *v*) reflects the influence of magnetic pressure on the material’s deformation behavior. This behavior underscores the significant role of magnetic fields in modulating stress distribution and deformation in nonlocal, rotating, and anisotropic media—an effect particularly important in high-performance materials and devices operating under magnetic and thermal environments, such as sensors, actuators, and aerospace components. When comparing the magnitude of the physical quantities for three different values of ${H}_{0}$, we found the fact that the effect of a magnetic field corresponds to the term signifying positive forces that tend to accelerate the metal particles. This is well in agreement with the physical situation and consistent with the results obtained by Das et al.^32^.

## Conclusion

The primary objective of this research is to construct a mathematical model that captures the behavior of normal stress, normal displacement, and temperature in an infinite orthotropic rotating generalized thermoelastic medium subjected to nonlocal effects, thermal loading, and a magnetic field within the framework of Green and Naghdi’s theory of type III. By applying the normal mode analysis method, we derive exact solutions without the need for assumptions on field variables. Numerical simulations are conducted using material properties resembling cobalt, with theoretical findings illustrated through graphical representations.

The combined theoretical and numerical analysis leads to the following key conclusions:

- The normal mode technique is adopted in the area of thermoelasticity and applied to those particular problems in which the coupled relationships between stress, strain, and temperature exists. This method provides accurate solutions without any pre- summed limitations on the physical variables that are present in the field equations. The normal mode technique is beneficial to solve problems in different fields, such as hydrodynamics and thermoelasticity.
- A two-dimensional orthotropic rotating thermoelastic model influenced by thermal load, nonlocal effects, and a magnetic field can be effectively described by a system of linear partial differential equations.
- All field variables remain continuous and satisfy the given boundary conditions, confirming that solid deformation is influenced by both the applied load and boundary constraints.
- The magnitude of all physical quantities increases with time, and their behavior remains consistent across different time values.
- The nonlocal parameter has a noticeable impact on the distribution and magnitude of all field variables.
- Field variables show strong sensitivity to rotational effects.
- The magnetic field significantly influences all the physical quantities under study.
- The validity of the generalized thermoelastic theory is supported by the fact that physical quantities are confined to a finite spatial region and decay to zero as distance increases, indicating a return to equilibrium.
- The insights presented in this study offer valuable contributions to researchers in material science, physicists, and designers of advanced materials, as well as those working on the development of hyperbolic thermoelastic theories. Moreover, the analytical approach employed here is broadly applicable to challenges in thermodynamics, thermoelasticity, and poroelasticity.

## Data Availability

The datasets used and/or analysed during the current study available from the corresponding author on reasonable request.

## Appendix A

$$\lambda _{1} = \frac{{\sqrt {\frac{\begin{gathered} 2 \times 2^{{1/3}} {\mathrm{m}}_{{11}}^{2} - 6 \times 2^{{1/3}} {\mathrm{m}}_{{22}} - 2{\mathrm{m}}_{{11}} \left( { - 2{\mathrm{m}}_{{11}}^{3} + 9{\mathrm{m}}_{{11}} {\mathrm{m}}_{{22}} - 27{\mathrm{m}}_{{33}} + \sqrt { - 4\left( {{\mathrm{m}}_{{11}}^{2} - 3{\mathrm{m}}_{{22}} } \right)^{3} + \left( {2{\mathrm{m}}_{{11}}^{3} - 9{\mathrm{m}}_{{11}} {\mathrm{m}}_{{22}} + 27{\mathrm{m}}_{{33}} } \right)^{2} } } \right)^{{1/3}} \hfill \\ \quad\quad + 2^{{2/3}} \left( { - 2{\mathrm{m}}_{{11}}^{3} + 9{\mathrm{m}}_{{11}} {\mathrm{m}}_{{22}} - 27{\mathrm{m}}_{{33}} + \sqrt { - 4\left( {{\mathrm{m}}_{{11}}^{2} - 3{\mathrm{m}}_{{22}} } \right)^{3} + \left( {2{\mathrm{m}}_{{11}}^{3} - 9{\mathrm{m}}_{{11}} {\mathrm{m}}_{{22}} + 27{\mathrm{m}}_{{33}} } \right)^{2} } } \right)^{{2/3}} \hfill \\ \end{gathered} }{{\left( { - 2{\mathrm{m}}_{{11}}^{3} + 9{\mathrm{m}}_{{11}} {\mathrm{m}}_{{22}} - 27{\mathrm{m}}_{{33}} + \sqrt { - 4\left( {{\mathrm{m}}_{{11}}^{2} - 3{\mathrm{m}}_{{22}} } \right)^{3} + \left( {2{\mathrm{m}}_{{11}}^{3} - 9{\mathrm{m}}_{{11}} {\mathrm{m}}_{{22}} + 27{\mathrm{m}}_{{33}} } \right)^{2} } } \right)^{{1/3}} }}} }}{{\sqrt 6 }},$$

$$\begin{gathered} \lambda _{2} = \frac{1}{{2\sqrt 3 }}\left( {\sqrt {} \left( {\left( {2{\mathbbm{i}}2^{{1/3}} ({\mathbbm{i}} + \sqrt 3 ){\mathrm{m}}_{{11}}^{2} + 2^{{1/3}} \times (6 - 6{\mathbbm{i}}\sqrt 3 ){\mathrm{m}}_{{22}} - 4{\mathrm{m}}_{{11}} \left( { - 2{\mathrm{m}}_{{11}}^{3} + 9{\mathrm{m}}_{{11}} {\mathrm{m}}_{{22}} - 27{\mathrm{m}}_{{33}} } \right.} \right.} \right.} \right. \hfill \\ \quad \quad \left. { + 3\sqrt 3 \sqrt { - {\mathrm{m}}_{{11}}^{2} {\mathrm{m}}_{{22}}^{2} + 4{\mathrm{m}}_{{22}}^{3} + 4{\mathrm{m}}_{{11}}^{3} {\mathrm{m}}_{{33}} - 18{\mathrm{m}}_{{11}} {\mathrm{m}}_{{22}} {\mathrm{m}}_{{33}} + 27{\mathrm{m}}_{{33}}^{2} } } \right)^{{1/3}} \hfill \\ \left. {\quad \quad + \left( { - 1 - {\mathbbm{i}}\sqrt 3 } \right)\left( { - 4{\mathrm{m}}_{{11}}^{3} + 18{\mathrm{m}}_{{11}} {\mathrm{m}}_{{22}} - 54{\mathrm{m}}_{{33}} + 6\sqrt 3 \sqrt { - {\mathrm{m}}_{{11}}^{2} {\mathrm{m}}_{{22}}^{2} + 4{\mathrm{m}}_{{22}}^{3} + 4{\mathrm{m}}_{{11}}^{3} {\mathrm{m}}_{{33}} - 18{\mathrm{m}}_{{11}} {\mathrm{m}}_{{22}} {\mathrm{m}}_{{33}} + 27{\mathrm{m}}_{{33}}^{2} } } \right)^{{2/3}} } \right)/ \hfill \\ \left. {\left. {\quad \quad \left( { - 2{\mathrm{m}}_{{11}}^{3} + 9{\mathrm{m}}_{{11}} {\mathrm{m}}_{{22}} - 27{\mathrm{m}}_{{33}} + 3\sqrt 3 \sqrt { - {\mathrm{m}}_{{11}}^{2} {\mathrm{m}}_{{22}}^{2} + 4{\mathrm{m}}_{{22}}^{3} + 4{\mathrm{m}}_{{11}}^{3} {\mathrm{m}}_{{33}} - 18{\mathrm{m}}_{{11}} {\mathrm{m}}_{{22}} {\mathrm{m}}_{{33}} + 27{\mathrm{m}}_{{33}}^{2} } } \right)^{{1/3}} } \right)} \right), \hfill \\ \end{gathered}$$

$$\begin{gathered} \lambda _{3} = \frac{1}{{2\sqrt 3 }}\left( {\sqrt {} \left( {\left( { - 2{\mathbbm{i}}2^{{1/3}} \left( { - {\mathbbm{i}} + \sqrt 3 } \right){\mathrm{m}}_{{11}}^{2} + 2^{{1/3}} \times \left( {6 + 6{\mathbbm{i}}\sqrt 3 } \right){\mathrm{m}}_{{22}} - 4{\mathrm{m}}_{{11}} \left( { - 2{\mathrm{m}}_{{11}}^{3} + 9{\mathrm{m}}_{{11}} {\mathrm{m}}_{{22}} - 27{\mathrm{m}}_{{33}} } \right.} \right.} \right.} \right. \hfill \\ \left. {\quad \quad + 3\sqrt 3 \sqrt { - {\mathrm{m}}_{{11}}^{2} {\mathrm{m}}_{{22}}^{2} + 4{\mathrm{m}}_{{22}}^{3} + 4{\mathrm{m}}_{{11}}^{3} {\mathrm{m}}_{{33}} - 18{\mathrm{m}}_{{11}} {\mathrm{m}}_{{22}} {\mathrm{m}}_{{33}} + 27{\mathrm{m}}_{{33}}^{2} } } \right)^{{1/3}} \hfill \\ \left. {\quad \quad + {\mathbbm{i}}\left( {{\mathbbm{i}} + \sqrt 3 } \right)\left( { - 4{\mathrm{m}}_{{11}}^{3} + 18{\mathrm{m}}_{{11}} {\mathrm{m}}_{{22}} - 54{\mathrm{m}}_{{33}} + 6\sqrt 3 \sqrt { - {\mathrm{m}}_{{11}}^{2} {\mathrm{m}}_{{22}}^{2} + 4{\mathrm{m}}_{{22}}^{3} + 4{\mathrm{m}}_{{11}}^{3} {\mathrm{m}}_{{33}} - 18{\mathrm{m}}_{{11}} {\mathrm{m}}_{{22}} {\mathrm{m}}_{{33}} + 27{\mathrm{m}}_{{33}}^{2} } } \right)^{{2/3}} } \right)/ \hfill \\ \left. {\left. {\quad \quad \left( { - 2{\mathrm{m}}_{{11}}^{3} + 9{\mathrm{m}}_{{11}} {\mathrm{m}}_{{22}} - 27{\mathrm{m}}_{{33}} + 3\sqrt 3 \sqrt { - {\mathrm{m}}_{{11}}^{2} {\mathrm{m}}_{{22}}^{2} + 4{\mathrm{m}}_{{22}}^{3} + 4{\mathrm{m}}_{{11}}^{3} {\mathrm{m}}_{{33}} - 18{\mathrm{m}}_{{11}} {\mathrm{m}}_{{22}} {\mathrm{m}}_{{33}} + 27{\mathrm{m}}_{{33}}^{2} } } \right)^{{1/3}} } \right)} \right). \hfill \\ \end{gathered}$$

## List of symbols

$$\rho$$: Material density
$${T}_{^\circ }$$: Absolute temperature
$$u,v$$: Displacement components
$${c}_{e}$$: Specific heat
$${c}_{11},{c}_{12},{c}_{22},{c}_{44}$$: Material’s constants
${H}_{0}$: The magnetic field
i: The imaginary unit number
$T$: The thermodynamics temperature
$${L}_{N}$$: Nonlocal parameter
${\mu }_{e}$: The magnetic permeability
*K*, *K*^***^: Constants
${\beta }_{1}{, \beta }_{2}$: Thermal coupling quantities
$$\Omega$$: Angular velocity
$$t$$: Time
$${\sigma }_{ij}$$: Stress components
$$b$$: Wave number
$$\omega$$: Angular frequency
